# Individual tree segmentation of airborne and UAV LiDAR point clouds based on the watershed and optimized connection center evolution clustering

**DOI:** 10.1002/ece3.10297

**Published:** 2023-07-12

**Authors:** Yi Li, Donghui Xie, Yingjie Wang, Shuangna Jin, Kun Zhou, Zhixiang Zhang, Weihua Li, Wuming Zhang, Xihan Mu, Guangjian Yan

**Affiliations:** ^1^ State Key Laboratory of Remote Sensing Science, Beijing Engineering Research Center for Global Land Remote Sensing Products Beijing Normal University Beijing China; ^2^ CESBIO University of Toulouse Toulouse France; ^3^ School of Geospatial Engineering and Science Sun Yat‐Sen University Zhuhai China

**Keywords:** airborne LiDAR scanning, connection center evolution, individual tree segmentation, unmanned aerial vehicle LiDAR scanning, watershed

## Abstract

Light detection and ranging (LiDAR) data can provide 3D structural information of objects and are ideal for extracting individual tree parameters, and individual tree segmentation (ITS) is a vital step for this purpose. Various ITS methods have been emerging from airborne LiDAR scanning (ALS) or unmanned aerial vehicle LiDAR scanning (ULS) data. Here, we propose a new individual tree segmentation method, which couples the classical and efficient watershed algorithm (WS) and the newly developed connection center evolution (CCE) clustering algorithm in pattern recognition. The CCE is first used in ITS and comprehensively optimized by considering tree structure and point cloud characteristics. Firstly, the amount of data is greatly reduced by mean shift voxelization. Then, the optimal clustering scale is automatically determined by the shapes in the projection of three different directions. We select five forest plots in Saihanba, China and 14 public plots in Alpine region, Europe with ULS or ALS point cloud densities from 11 to 3295 pts/m^2^. Eleven ITS methods were used for comparison. The accuracy of tree top detection and tree height extraction is estimated by five and two metrics, respectively. The results show that the matching rate (*R*_match_) of tree tops is up to 0.92, the coefficient of determination (*R* ^2^) of tree height estimation is up to .94, and the minimum root mean square error (RMSE) is 0.6 m. Our method outperforms the other methods especially in the broadleaf forests plot on slopes, where the five evaluation metrics for tree top detection outperformed the other algorithms by at least 11% on average. Our ITS method is both robust and efficient and has the potential to be used especially in coniferous forests to extract the structural parameters of individual trees for forest management, carbon stock estimation, and habitat mapping.

## Graphical abstract

We propose a new hybrid individual tree segmentation method, which couples the classical and efficient watershed algorithm and the newly developed connection center evolution clustering algorithm in pattern recognition. Our method is both robust and efficient and has the potential to be used for complex forests to extract the structural parameters of individual trees for forest management, carbon stock estimation, and habitat mapping.

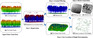

## 1 INTRODUCTION

Forests, as a vital part of terrestrial ecosystems, play an important role in global climate change and biodiversity (Liang et al., 2016; Seidl et al., 2017). It is challenging to conduct resource surveys of forests, especially at the individual tree scale. In the past, forest resource surveys often relied on field measurements, which were time‐consuming and laborious. In recent years, remote sensing data have been increasingly applied to forestry. 2D optical images have been used to estimate forest morphological parameters (e.g., canopy cover and leaf area index) (Korhonen et al., 2017). However, these data are unable to retrieval three dimensional (3D) structural information of trees (Zheng et al., 2021). Light detection and ranging (LiDAR) data provide 3D structural information of objects and are ideal for extracting individual tree parameters of forests (Lefsky et al., 2002). There are two main categories of LiDAR for extracting individual tree parameters: ground‐based and air‐based. Ground‐based LiDAR, such as terrestrial LiDAR scanning (TLS), has a high distance accuracy of the measurement and denser points within the limited extent, which is suitable for delicate structural parameter extraction at the plot scale (Burt et al., 2019; Tao et al., 2015). Air‐based LiDAR including airborne LiDAR scanning (ALS) and unmanned aerial vehicle LiDAR scanning (ULS) can be applied to survey 3D information in a bigger region than TLS with a little lower points density. Considering that ALS and ULS can acquire the 3D structural characteristics of trees on a large scale in complex terrain conditions, they are often used in forest survey (Guo et al., 2020).

Individual tree segmentation (ITS) also known as individual tree detection (ITC) or individual tree and crown delineation (ITCD) from point clouds generated via ALS or ULS is a considerable challenge (Lindberg & Holmgren, 2017). There are mainly three categories of methods for ITS based on ALS data, including raster‐based methods, point‐based methods, and joint methods. The raster‐based methods first convert 3D point clouds into 2D rasters, such as canopy height models (CHMs) or digital surface models (DSMs), and then use image processing or computer vision techniques for ITS. Specific algorithms include the watershed (Jing et al., 2012; Wang et al., 2004), region growing (Dalponte & Coomes, 2016; Solberg et al., 2006), valley following (Katoh & Gougeon, 2012; Leckie et al., 2005), marker‐controlled watershed (Chen et al., 2006; Hu et al., 2014), variable window filtering (Hyyppa et al., 2001), mean‐shift clustering (Dai et al., 2018), and graph‐cut (Strîmbu & Strîmbu, 2015) algorithms. These methods are usually more efficient, but the part of the information will inevitably be lost when the 3D point clouds are converted into 2D rasters (Zhen et al., 2016). In addition, CHMs or DSMs may also have pits, which dramatically affect the accuracy of the segmentation algorithm (Yang et al., 2019; Zhang et al., 2020). The point‐based methods directly utilize primitive or voxelized point clouds for ITS, such as point cloud region growing (Li et al., 2012; Lu et al., 2014), layer stacking (Ayrey et al., 2017), *k*‐means (Lindberg et al., 2014), and graph cut (Lindberg et al., 2014; Williams et al., 2019). These methods can better use the 3D structure information of the point cloud data and further improve segmentation accuracy (Zhen et al., 2016). However, these methods also suffer from complex parameters, poor generalizability, or low efficiency. The joint methods combine the first two in the hope of achieving a better result. For example, Tochon et al. (2015) combined the watershed and *k*‐means algorithms to ITS in conifer and broadleaf forests. Reitberger et al. (2009) first extracted the trunk using the watershed algorithm and then used the extracted trunk as a priori knowledge of normalized cut. The joint methods combine the advantages of the first two categories of methods and therefore can improve the segmentation accuracy, but will also inherit both the disadvantage of the raster‐ and point cloud‐based methods. In some studies, data from ALS and ULS have not been distinguished because of the similarity of their data collection principles (Yun et al., 2021). But in fact they differ significantly in point density. The point density of ALS is typically limited to 10 points/m^2^, while the point density of ULS can range from 10 to t1000 points/m^2^ depending on the flight altitude and sensor characteristics (Kellner et al., 2019; Lu et al., 2014). As a result, ULS usually contains more detailed information than ALS. ITS studies for ULS have been conducting to achieve better segmentation result. For example, Wallace et al. (2014), Balsi et al. (2018) and Yin and Wang (2019) used ULS for ITS in homogenous forest. Jaskierniak et al. (2021) develop a bottom‐up approach of ITS for mixed species eucalypt forests. Although these studies have get good results, the forest scenes are homogenous or specific.

Several critical issues about the presented ITS methods of ALS and ULS are summarized as follows: (1) There is an urgent need to propose more general and flexible methods that are not specific to data sources or forest types. Vauhkonen et al. (2012) compared six different ITS methods and found that the forest structure strongly affected the performance of all algorithms. Wang et al. (2016) found that point density was a highly influential factor in the performance of the methods that use point cloud data. Robust methods that are not sensitive to point density (both suit for ALS and ULS) and can be applied to coniferous, broadleaf, and mixed forests are rarely seen in the current studies. (2) There is an urgent need to propose methods that are specific to certain challenging forest types or scenarios. Dense vegetation, undulating terrain, differences in canopy shape and size, etc. can make it difficult to ITS. It is necessary to analyze the mechanism of the impact of special scenarios on ITS and propose targeted solutions. For example, the issue of omission (under‐segmentation) is a big challenge for most ITS methods for dense forests (Table 1). A summary about under‐ and over‐segmentation percentages of some ITS methods is listed in Table 1. According to the study of Li et al. (2012), when the tree stem density increases from 0.05 to 0.07 trees/m^2^, the percentage of omission greatly increases from 15% to 29% even in conifer forests. Broadleaf and mixed forests even have bigger omission fractions than conifers because of the complex structures and various species of trees. The reason for these results is that there is a severe mutual shading effect among the trees in the dense forest. Therefore, methods that make full use of the detailed information in the point cloud are needed.

**TABLE 1:** Segmentation accuracy of several ITS methods affected by tree types and density.

Algorithms	Type	Density (trees/m^2^)	Matched (%)	Omitted (%)	Committed (%)	Reference
Point Cloud Region Growing	Conifer	0.05	85	15	0	Li et al. (2012)
		0.06	74	26	0	
		0.07	71	29	0	
Marker‐controlled Watershed	Deciduous trees & Conifer	Unknown	74	26	8	Hu et al. (2014)
Point‐based Algorithm	Mixed mountainous forest	0.02	75	25	12	Véga et al. (2014)
	Conifer	0.02	93	7	2	
	Broadleaf	0.05	80	20	14	
Bottom‐up Region Growing	Deciduous‐ broadleaf	0.02	84	16	3	Lu et al. (2014)
Marker‐controlled Watershed	Broadleaf	Unknown	~70	~30	0	Zheng et al. (2021)

*Note*: Matched (%) = the number of correctly segmented trees/the number of trees in plots; Omitted (%) = the number of under‐segmented trees/the number of trees in plots; Committed (%) = the number of over‐segmented trees/the number of trees in plots.

Joint ITS methods take the advantages of both the high efficiency of the raster‐based methods and the high accuracy of the point‐based methods, which have better development prospects. The basic idea of the joint methods is to use the raster‐based methods for initial segmentation and then the point‐based methods for fine segmentation. Many point clustering algorithms in pattern recognition can be used for fine segmentation, such as *k*‐means (Lindberg et al., 2014), mean‐shift (Dai et al., 2018), and graph‐based algorithms (Lindberg et al., 2014; Williams et al., 2019). However, these algorithms directly rely on the input parameters, and different parameters may yield very different results (Geng & Tang, 2020). Therefore, it is necessary to develop a robust clustering algorithm that does not depend excessively on the input parameters.

In this study, we propose a new joint individual tree segmentation algorithm coupled with the watershed and optimized connection center evolution algorithm. Firstly, we use a pit‐free canopy height model to implement initial segmentation based on the watershed (WS) algorithm, which has the advantages of high efficiency. Secondly, we introduce a new clustering algorithm called connection center evolution (CCE), which extends the concept of the number of paths in graph theory to the case of arbitrary real numbers and can automatically skip the unreasonable number of clusters (Geng & Tang, 2020). and then fine segmentation based on the optimized CCE algorithm, which reduced data amount by voxelization and determines the optimal clustering scale by different planar projections.

The motivation of this study is to provide individual tree attributes such as height and location for the construction of large‐scale digital forestry. Therefore, a general and efficient ITS method is expected. For this purpose, ALS and ULS data from different forest types, such as coniferous, broadleaf and mixed forests, with different point cloud densities were used and validated by location and tree height. This paper is organized according to the following structure. In Section 1, we introduce the overview of our study site and datasets and describe how the data are preprocessed. The basic principle and framework of our method are explained in Section 2. In Section 3, the results and analysis are displayed. The discussion and conclusion are explained in Sections 4 and 5, respectively.

## 2 MATERIALS AND METHODS

### 2.1 Study site and datasets

#### 2.1.1 Study area

Our study plots are located in Saihanba National Forest Park, China (42°28′54″ N, 117°16′28″ E). The vegetation types are varied, and the main forest types include deciduous coniferous forests, evergreen coniferous forests, mixed coniferous forests, and broadleaf forests. The major tree species include the larch trees (*Pincus sylvestris* var. *mongolica* Litv.), Mongolian pine (*Larix principis‐rupprechtii* Mayr), and birch trees (*Betula platyphylla*). Saihanba National Forest Park is a multifunctional botanical park integrating scientific research and plant species collection.

#### 2.1.2 Plots

**FIGURE 1:**
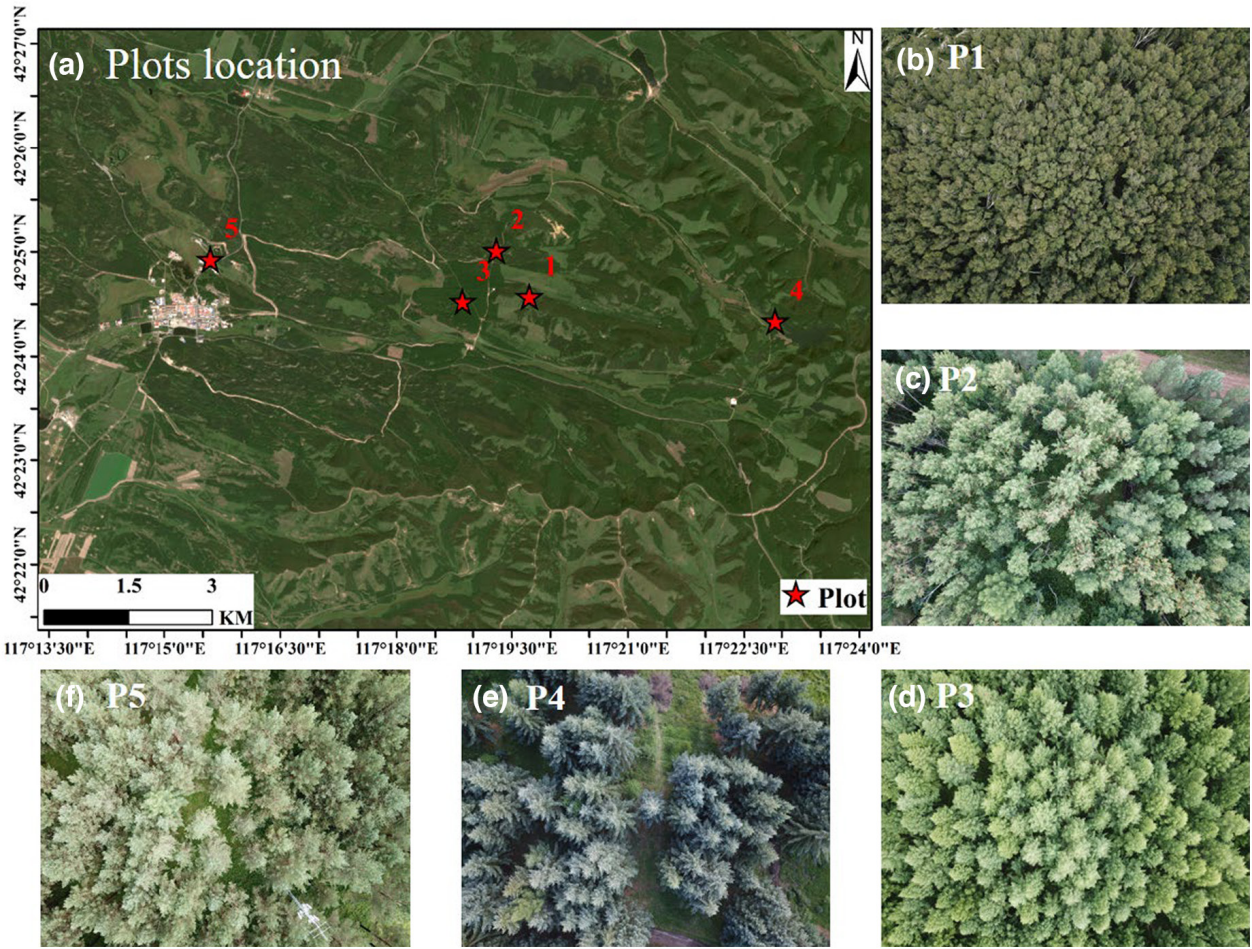
Plots location in the study area (a). The tree types of five plots (P1–P5) are birch (b), mixed (c), larch (d), spruce (e), and Mongolian pine (f), respectively. All plots photographs are clipped from UAV RGB images.

We selected five forest plots for the validation (Figure 1). P1 is a deciduous broadleaf forest plot (birch); P2 is a mixed forest plot containing deciduous coniferous and evergreen coniferous and deciduous broadleaf (mixed with aspen, larch, Mongolian pine, spruce, and birch); P3 is a deciduous coniferous forest plot (larch); P4 and P5 are both evergreen coniferous forests (including spruce and Mongolian pine, respectively). The area of the plots is 30 m × 30 m or 50 m × 50 m, and the average tree density of all 5 plots is 0.10 trees/m^2^. The specific information of these plots is shown in Table 2.

**TABLE 2:** Characteristics of five forest plots.

Plot	Tree type	Average height (m)	Number of trees	Stem density (trees/m^2^)	Point density (pts/m^2^)	Size (m^2^)
P1	Birch	15	122	0.14	298	30 × 30
P2	Mixed	18	89	0.10	3295	30 × 30
P3	Larch	21	121	0.05	1636	50 × 50
P4	Spruce	15	87	0.10	1473	30 × 30
P5	Mongolian pine	16	87	0.10	3976	30 × 30

### 2.2 Data acquisition and preprocessing

The data in this study include both point cloud data and field measurement data. Point cloud data in each plot were obtained by ULS and TLS devices. ULS data were used to test the ITS methods, while the combination of TLS and field measurement data is used to obtain accurate reference locations for each tree. It is extremely difficult to measure the height of a large number of single trees in the field, especially for our study area where the tree height is usually greater than 10 m. Therefore, we merged the ULS and TLS data and then manually extracted the tree height of each tree as reference. The reference tree height and location were also used to evaluate the ITS methods.

#### 2.2.1 Acquisition of LiDAR and field data

The specific data include the following three types. (1) ULS point clouds: The ULS data were obtained in July 2022 using RIEGL VUX‐1UAV mounted on the DJI M600 platform. The drone flies at an altitude of approximately 50–200 m based on the topography and tree height of the different plots. The specific ULS point cloud densities of each plot are shown in Table 2. (2) TLS point clouds: A Riegl VZ‐1000 terrestrial laser scanner was used to obtain multi‐station scanning data at the sampling center and corners in order to relieve the occlusion issue. Depending on plot size and canopy characteristics, 9–17 scanning stations were set up. (3) Field data: Fieldwork was also carried out in August 2022. We used HI TARGET Qstar 8 Mobile GPS to locate the center points of the plots. The location of each tree in all plots was checked and corrected by manual field surveys according to tree locations extracted from TLS (see Section 2.2.2). We did not use GPS to locate each tree because of the large uncertainty in positioning in the understory.

#### 2.2.2 Data preprocessing

**FIGURE 2:**
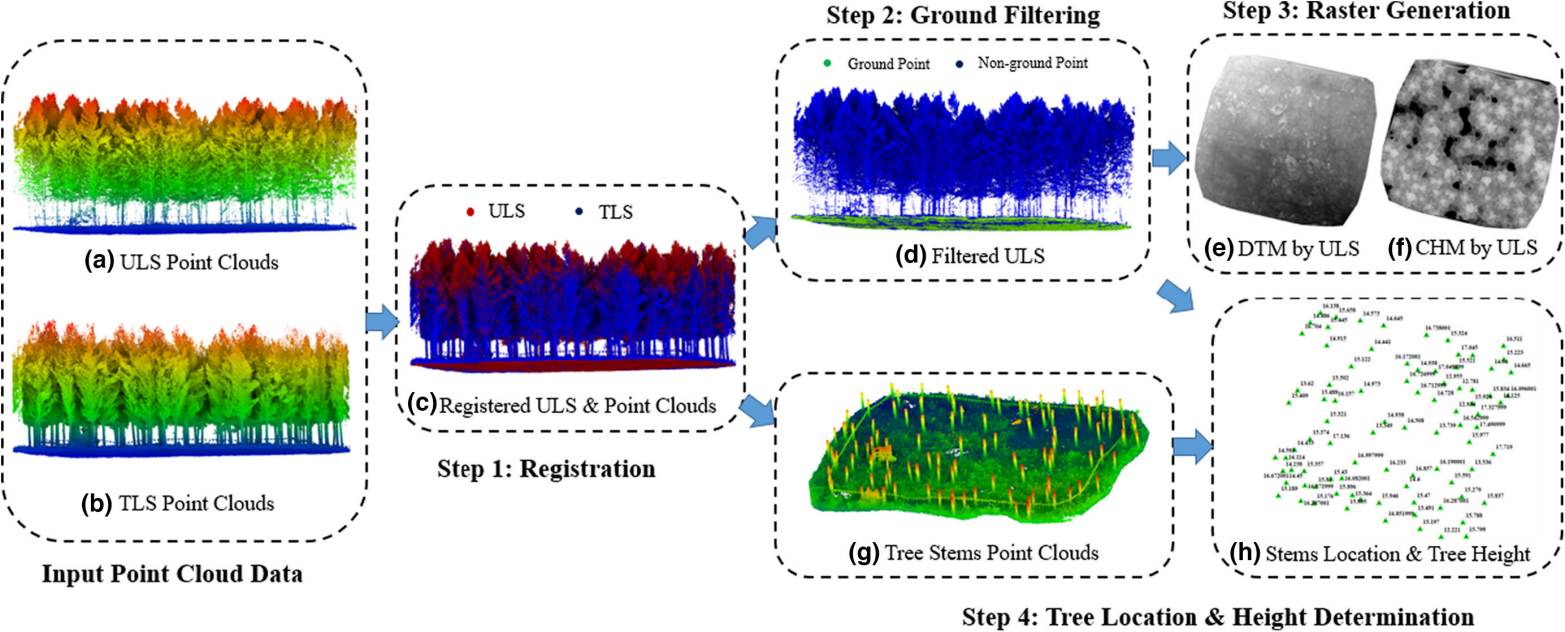
Data preprocessing procedure including (1) Registration; (2) Ground Filtering; (3) Raster Generation; (4) Tree Location and Height Determination.

Data preprocessing for the ULS, TLS, and field trunk position data includes the four‐step operations, which are illustrated intuitively in Figure 2. (1) Registration: ULS and TLS data were manually registered with each other to avoid positioning bias between these two datasets by manually selecting control points (Figure 2c). (2) Ground filtering: The cloth simulation filter (CSF) proposed by Zhang et al. (2016) was used to separate ground and nonground point clouds. The ULS filtering results are shown in Figure 2d. (3) Raster generation: After filtering, DTM (Digital Terrain Model) and CHM were generated using lidR tools (Roussel et al., 2020). Grid resolutions were set to 0.05 or 0.1 m for ULS data according to the point density in the specific plots. The Delaunay triangulation (TIN) algorithm was used for spatial interpolation and DTM generation (Axelsson, 2000). The pit‐free algorithm developed by Khosravipour et al. (2014) was used to generate pit‐free CHMs (see Section 2.4.1). These CHMs are used as the input of the algorithm, and DTMs are used to normalize the ULS point clouds. (4) Tree Location & Height Determination: TLS data were segmented with a height threshold value of approximately 1.5 m. Only point clouds less than 1.5 m were kept. After that, tree stems could be seen clearly through segmented TLS data (Figure 2g). Then, tree location was corrected by fieldwork according to extracted tree stems. Finally, according to the corrected tree stems distribution, each tree height was measured manually using ULS point cloud. The obtained positions and tree heights were used for the validation of the individual tree segmentation algorithms as detailed in the results section.

### 2.3 Benchmark airborne LiDAR point clouds

To demonstrate the applicability of the ITS method in different study areas, forest types, and point cloud densities, we used a benchmark airborne LiDAR point cloud dataset with individual tree inventory data in the Alpine Space, Europe (Eysn et al., 2015). This dataset includes 14 different plots located in four European countries and can be downloaded from the NEWFOR website (https://www.newfor.net/). The detailed descriptions of these plots are shown in Table 3. Due to the low point cloud densities in these plots, the resolutions of CHMs and DTMs generated by ALS point clouds were set to 0.2 m.

**TABLE 3:** Characteristics of 14 forest plots in the Alpine Space of Europe. The plot numbers are discontinuous because the data of 05, 12, 13, and 14 plots are not available.

Plot	Tree type	Average height (m)	Number of trees	Stem density (trees/m^2^)	Point density (pts/m^2^)	Size (m^2^)	Study area
01	Fir, beech	17	359	0.04	13	10,000	Saint‐Agnan, France
02	Scots pine, larch, spruce	18	106	0.08	11	1300	Cotolivier, Italy
03	Scots pine, larch	17	49	0.04			
04	Larch, sycamore	13	22	0.02			
06	Spruce	14	107	0.04	22	3000	Montafon, Austria
07	Spruce, larch, fir	16	49	0.04	95–121	1300	Asiago, Italy
08	Larch, spruce, fir, sycamore, poplar	14	235	0.19			
09	Spruce, fir	24	80	0.07	11		
10	Spruce, fir, beech	17	110	0.09			
11		14	183	0.13			
15	Fir, spruce, beech	23	53	0.03	30	2000	Leskova, Slovenia
16		25	37	0.02			
17	Fir, spruce, beech, sycamore, elm	21	117	0.06			
18	Fir, beech, sycamore	25	92	0.05			

### 2.4 The method

**FIGURE 3:**
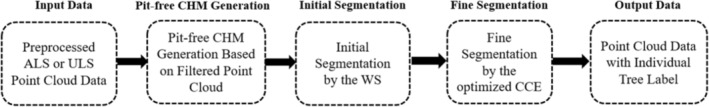
The implementation of the ITS method. Three steps are included: (1) Pit‐free CHM generation; (2) initial segmentation using the WS; (3) fine segmentation using the optimized CCE.

Our ITS method consists of three main components: pit‐free CHM generation, initial segmentation using the WS, and fine segmentation using the optimized CCE. The implementation is shown in Figure 3.

#### 2.4.1 Pit‐free CHM generation

The pit‐free CHM can eliminate the pits and thus reduce over‐segmentation (Yang et al., 2019). First, the nonground points are normalized according to the DTMs. Then, the normalized point clouds are horizontally segmented at 0, 2, 5, 10, and 15 m. For each segmented layer, multiple‐level CHMs are generated using the TIN algorithm according to the highest point. Finally, the pit‐free CHM is generated by taking the maximum value of these multiple‐level CHMs in the corresponding pixels (Khosravipour et al., 2014).

#### 2.4.2 Initial segmentation using the WS

**FIGURE 4:**
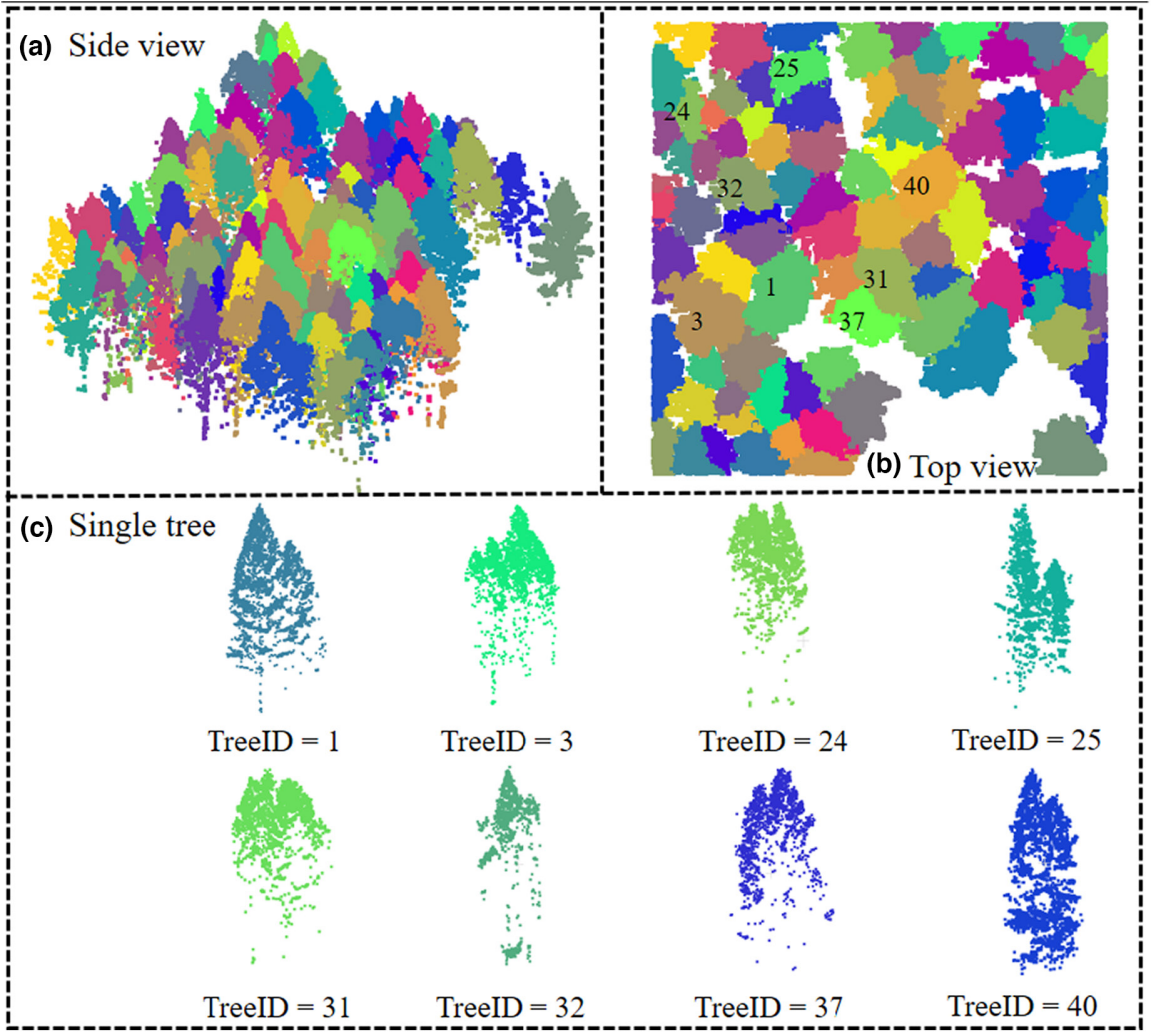
Initial segmentation of ULS point cloud data using WS. The results are shown in the side view of the plot (a), and in the top view (b), individually. The IDs and colors of segmented trees are randomly assigned. It is obviously suffered from omission issue. Therefore, eight under‐segmentation trees were taken as examples and manually selected in subplot (c).

The WS is an image region segmentation method, which takes the similarity with the neighboring pixels as an essential reference in the segmentation process so that the pixels with similar spatial locations and similar grayscale values (height value in the CHM) are connected to form a closed contour (Wang et al., 2004). Here, the lidR tools developed by Roussel et al. (2020) are used to implement the WS and get the initial segmentation results. There are two input parameters: height tolerance (denoted as *tolerance*) and neighborhood search radius (denoted as *ext*). *Tolerance* represents the minimum height of the object in the units of image intensity between its highest point (seed) and the point where it contacts another object (checked for every contact pixel). If the height is smaller than the *tolerance*, the object will be combined with one of its neighbors, which is the highest. *Ext* represents the radius of the neighborhood in pixels for the detection of neighboring objects. A higher *ext* value smoothes out small objects. Figure 4 shows an example of the ITS results by the WS. Consistent with Table 1, the method suffers from significant under‐segmentation.

#### 2.4.3 Fine segmentation using the optimized CCE

In this part, the CCE algorithm is optimized and used for fine segmentation. The CCE first constructs the similarity matrix between each point, then performs the power multiplication operation on the similarity matrix continuously, and finally determines the aggregation center and the number of clusters by comparing the element sizes of the similarity matrix after each power operation. The CCE is considered an efficient and elegant clustering algorithm in Pattern Recognition (Geng & Tang, 2020). Concepts such as the number of walks and undirected graph in graph theory are extended, and the implementation of the CCE involves only the matrix power operation and does not require any human intervention. It suggests appropriate observation scales and provides corresponding clustering results. Here, we extend this algorithm for ITS of LiDAR point clouds. However, there are two issues in the original CCE algorithm. First, it is challenging to be implemented on point clouds with a large amount of data because of heavy computations. Second, it does not specify how to determine the most appropriate observation scale. To this end, we optimized the CCE algorithm, which can greatly reduce the amount of data by mean shift voxelization and automatically determine the optimal observation scale by crown projection. This optimized CCE algorithm consists of four main steps: mean shift voxelization, similarity matrix construction, CCE clustering, and Automatic determination of optimal scale.

1. *Mean shift voxelization*. Mean shift is a nonparametric feature‐space mathematical analysis technique and has been used for cluster analysis in computer vision and image processing (Comaniciu & Meer, 2002). Pang et al. (2021) used this algorithm for irregular voxelization of ALS point clouds and achieved fast and robust results. A consistent voxelization program is adopt, and the amount of data is reduced by approximately a factor of 10.
2. *Similarity matrix construction*. For each “individual tree” point clouds that has been initially segmented and voxelized, we construct the point‐to‐point distance matrix *D*:

(1) $$D=\left[\begin{array}{ccccc} 0 & d_{1,2} & \ldots & d_{1,n-1} & d_{1,n}\\ d_{2,1} & 0 & \ldots & \ldots & d_{2,n}\\ \ldots & \ldots & \ldots & \ldots & \ldots \\ d_{n-1,1} & \ldots & \ldots & 0 & d_{n-1,n}\\ d_{n,1} & d_{n,2} & \ldots & d_{n,n-1} & 0 \end{array}\right]$$

where $d_{i,j}=\sqrt{n_{i}\times n_{j}\times {\left(x_{i}-x_{j}\right)}^{2}+{\left(y_{i}-y_{j}\right)}^{2}+\mathrm{Vr}{\left(z_{i}-z_{j}\right)}^{2}}$ represents the variable related to the distance between point *p*_*i*_ and point *p*_*j*_. *Vr* is the vertical distance correction factor (value range is 0–1), which is introduced to consider the incompleteness of the ULS/ALS point clouds in the lower part of the tree canopy due to occlusion. *n*_*i*_ and *n*_*j*_ are the weights of the two voxels, that are used to maintain the consistency of the voxel space with the original point clouds.

Next, the distance matrix (*D*) can be converted to the similarity matrix ($\tilde{S}$) by the Gaussian kernel function (Geng & Tang, 2020) as follows:

(2) $${\tilde{s}}_{i,j}=\exp\left(-d_{i,j}^{2}/\sigma^{2}\right)$$

(3) $$\tilde{S}=\left[\begin{array}{ccccc} {\tilde{s}}_{1,1} & {\tilde{s}}_{1,2} & \ldots & {\tilde{s}}_{1,n-1} & {\tilde{s}}_{1,n}\\ {\tilde{s}}_{2,1} & {\tilde{s}}_{2,2} & \ldots & \ldots & {\tilde{s}}_{2,n}\\ \ldots & \ldots & \ldots & \ldots & \ldots \\ {\tilde{s}}_{n-1,1} & \ldots & \ldots & {\tilde{s}}_{n-1,n-1} & {\tilde{s}}_{n-1,n}\\ {\tilde{s}}_{n,1} & {\tilde{s}}_{n,2} & \ldots & {\tilde{s}}_{n,n-1} & {\tilde{s}}_{n,n} \end{array}\right]$$

where *σ* is an empirical coefficient that controls the size of the Gaussian kernel function. The element ${\tilde{s}}_{i,j}$ represents the similarity between *p*_*i*_ and *p*_*j*_.

The similarity matrix is similar in concept to the adjacency matrix, but the elements of the similarity matrix can be real numbers. Typically, the elements themselves are the most similar, so the diagonal elements of the similarity matrix are maximal.

Finally, the similarity matrix needs to be normalized as follows:

(4) $$\left\{\begin{array}{l} S={\tilde{D}}^{-1/2}{\tilde{S}\tilde{D}}^{-1/2}\\ \tilde{D}=\mathrm{diag}\left(d_{1},d_{2},\ldots ,d_{n}\right)\\ d_{i}=\sum_{j=1}^{n}{\tilde{s}}_{\mathrm{ij}} \end{array}\right.$$

where $\tilde{D}$ is the degree matrix of *S* and *d*_*i*_ is the degree of the *i*th point (*p*_*i*_).

3 CCE clustering. First, the power operation is performed on the normalized similarity matrix to obtain the following *k*‐order connectivity:

(5) $$\left\{\begin{array}{l} S^{k}\\ s_{i,j}^{k} \end{array}\right.k=1,2,\ldots$$

The entry ($s_{i,j}^{k}$) of the *k*th power (*S* ^*k*^) of the similarity matrix (*S*) is defined as the *k*‐order connectivity between *p*_*i*_ and *p*_*j*_ (denoted as ${\mathrm{con}}^{\left(k\right)}\left(p_{i},p_{j}\right)$). In particular, the diagonal entry $s_{i,i}^{k}$ is defined as the *k*‐order connectivity of point $p_{i}$ (denoted as ${\mathrm{con}}^{\left(k\right)}\left(p_{i},p_{i}\right)$). For each *k*, the *k*‐order relative connectivity of all points can be calculated, and the clustering centers will be determined according to the following rules: If one point satisfies Equation (6), it will be a connection center of the graph and is defined as a *k*‐order clustering center of the data.

(6) $$s_{i,i}^{k}>s_{i,j}^{k},\;j=1,\ldots ,n\left(j\neq i\right)$$

After the clustering centers are determined, the relative connectivity (${\text{rcon}}^{\left(k\right)}\left(i,j\right)$) is calculated according to Equation (7), and the clustering rules ($p^{*}$) are determined according to Equation (8). If we have *m* clustering centers $p_{c_{i}}\left(c_{i}\in \left\{1,2,\ldots ,n\right\}\;\text{and}\;i=1,2,\ldots ,m\right)$, for any point *p*_*j*_, it will be assigned to p*, where p* satisfies Equation (8).

(7) $${\text{rcon}}^{\left(k\right)}\left(i,j\right)=s_{i,j}^{k}/s_{i,i}^{k}$$

(8) $$p^{*}=\underset{p_{c_{i}}}{\arg\max}\left({\text{rcon}}^{\left(k\right)}\left(p_{c_{i}},p_{j}\right)\right)$$

For some datasets, for different values of *k*, we may obtain the same clustered data but with slightly different clustering results. In this situation, we can retain the optimal clustering results by introducing the normalized cut as follows:

(9) $$\left\{\begin{array}{l} \text{Ncut}\left(P_{1},P_{2},\ldots ,P_{m}\right)=\sum_{l=1}^{m}\sum_{p_{i}\in P_{l},p_{j}\in \overset{-}{P_{l}}}s_{\mathrm{ij}}^{\left(k\right)}/\mathrm{Vol}\left(P_{l}\right)\\ \mathrm{Vol}\left(P_{l}\right)=\sum_{p_{i}\in P_{l},p_{j}\in P}s_{\mathrm{ij}}^{\left(k\right)} \end{array}\right.$$

where ${\bar{P}}_{l}$ represents the complement of *P*_*l*_ in *P* and $\mathrm{Vol}\left(P_{l}\right)$ is the sum of *k*‐order connectivity between all points in *P*_*l*_ and all points in *P*.

**FIGURE 5:**
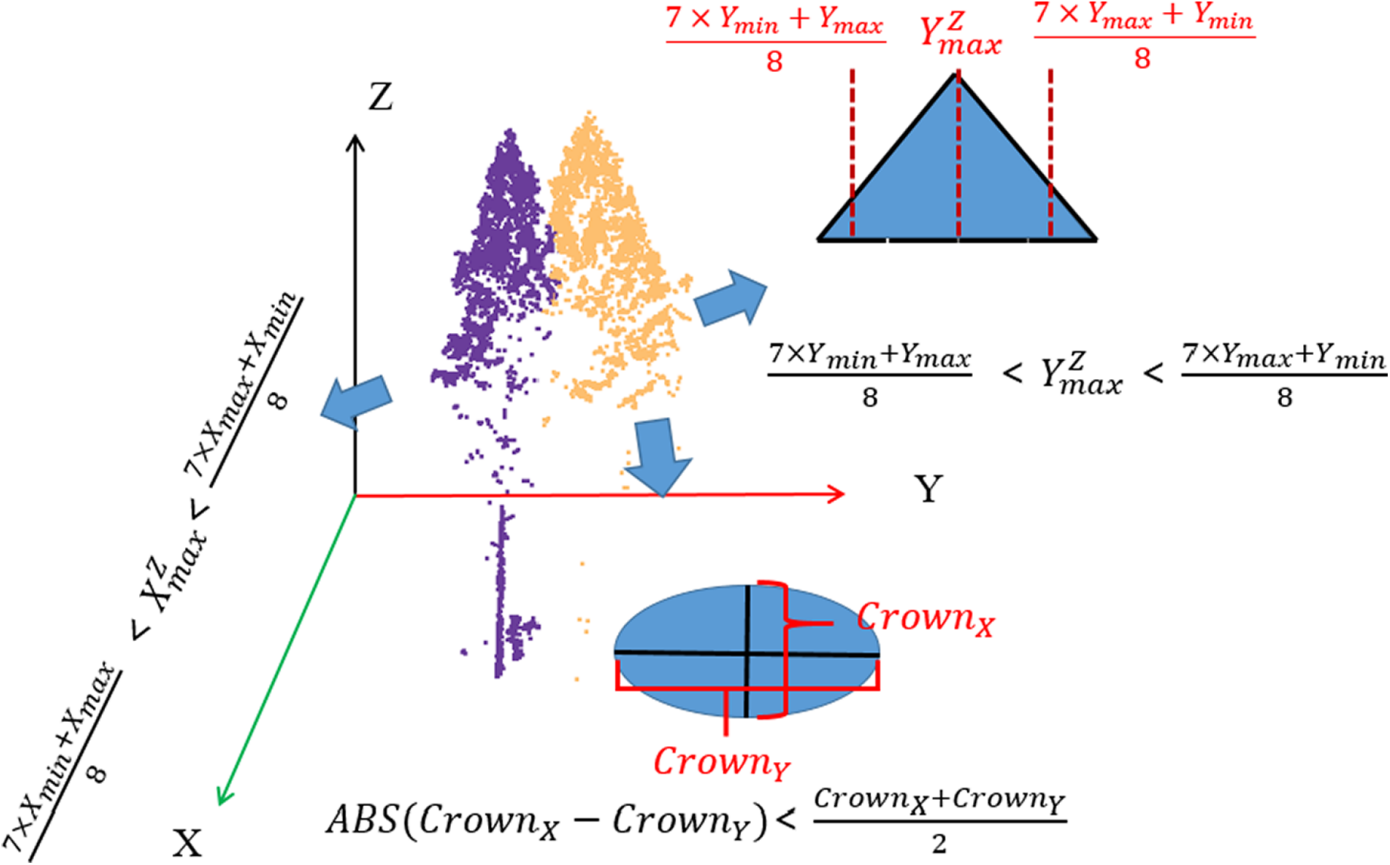
Diagram of segmented point clouds projected to *X–Y*, *X–Z*, and *Y–Z* planes. The blue filled graph shows the approximate outline of the tree point cloud projected onto the *X*–*Y* and *Y*–*Z* planes. ${\text{Crown}}_{X}$ and ${\text{Crown}}_{Y}$ are the crown widths along the *X*‐axis and *Y*‐axis direction. $X_{\max}^{Z}$ is the *x* value of the point with maximum *z* projected onto the *X–Z* plane. $Y_{\max}^{Z}$ is the corresponding parameter on the *Y–Z* plane. Three constraint inequalities are list on Corresponding projection planes.

4 *Automatic determination of optimal scale*. According to the CCE clustering, the clustering situation of different scales can be determined. When *k* = 1, each point is a clustering center, which is the most microscopic case. As the value of *k* increases, more points will be grouped together, which is the macroscopic case. We need to determine that the clustering results of the optimal scale and correctly segment individual trees. For this purpose, we project each scale of clustering result point clouds to the *X–Y*, *X–Z* and *Y–Z* plane, respectively (Figure 5). Then, we determine whether the following three inequalities hold in each of the three projection planes:

(10) $$\mathrm{ABS}\left({\text{Crown}}_{X}-{\text{Crown}}_{Y}\right)<\frac{{\text{Crown}}_{X}+{\text{Crown}}_{Y}}{2}\;\left(X-Y\;\text{Plane}\right)$$

(11) $$\frac{7\times X_{\min}+X_{\max}}{8}<X_{\max}^{Z}<\frac{7\times X_{\max}+X_{\min}}{8}\;\left(X-Z\;\text{Plane}\right)$$

(12) $$\frac{7\times Y_{\min}+Y_{\max}}{8}<Y_{\max}^{Z}<\frac{7\times Y_{\max}+Y_{\min}}{8}\;\left(Y-Z\;\text{Plane}\right)$$

${\text{Crown}}_{X}$ and ${\text{Crown}}_{Y}$ are the crown widths along the *X*‐axis and *Y*‐axis direction. $X_{\max}^{Z}$ is the *x* value of the point with maximum *z* projected onto the *X*–*Z* plane, $X_{\min}$ and $X_{\max}$ are the maximum and minimum *x* values in all points projected to this plane, respectively. $Y_{\max}^{Z}$, $Y_{\min}$, and $Y_{\max}$ are the corresponding parameters on the *Y–Z* plane. Finally, we filter the clustering results that satisfy the above conditions. If there are multiple candidate results, the one with the maximum number of the candidates will be selected as the best. Equations ((10), (11), (12)) ensures that the segmented tree shape is rational. Equation (10) requires that the larger of the crown width in the *X* and *Y* directions does not exceed three times that of the smaller, and canopies that exceed this limit are rare in nature. The sensitivity of the parameters of Equations (11) and (12) is analyzed in Section 4.1.

The input parameters of our method are summarized in Table 4. In addition, for plantations with trees of relatively similar growth, we followed the postprocessing method proposed by Pang et al. (2021). If the distance between two adjacent individual trees is less than the average crown diameter of the corresponding plot and the elevation of these two trees is less than 10 m, they will be merged into an individual tree. The average crown width is calculated using the segmented point clouds by our ITS algorithm. The watershed algorithm and data processing also involve the corresponding parameters. The sensitivity analysis of these parameters is not addressed in this study, as it has been previously analyzed by corresponding studies (Pang et al., 2016; Wang et al., 2004). Finally, each tree height and location are automatically extracted by calculating the height and geographical coordinates of the highest point.

**TABLE 4:** Description of two input parameters in our ITS method.

Parameter	Description	Purpose
*Vr*	The vertical distance correction factor	Reducing the influence of incompleteness of the ULS/ALS point clouds in the lower part of the tree canopy due to occlusion.
σ	Empirical coefficient related to the Gaussian kernel	Controlling the size of the Gaussian kernel function when converting the distance matrix into the similarity matrix.

### 2.5 Accuracy assessment

LiDAR point clouds with tree labels are output after applying the ITS method. Then, horizontal location and tree height are matching to the field reference data. The matching method started from the highest detected tree and searched for the reference trees that satisfied the height and distance criterion as match candidates. If a farther candidate showed a better height difference, then it became a better match. This process was repeated until all detected trees have been checked. If the closest one with the smallest height difference is the matched detection tree previously, these two trees will be treated as a matched pair (Pang et al., 2021). The matching criterion is described by Eysn et al. (2015). Eventually, a series of matching parameters are calculated. TP (true positive) is the number of correctly segmented trees; FN (false negative) is the number of trees not segmented but assigned to a nearby tree (omission error or under‐segmentation); FP (false‐positive) is the number of trees that did not exist but were segmented from the point cloud (commission error or over‐segmentation).

We select extraction rate (*R*_extraction_), matching rate (*R*_match_), commission rate (*R*_commission_), omission rate (*R*_omission_), and F score (*F*) as evaluation metrics (Eysn et al., 2015; Li et al., 2012). Here are the expressions.

(13) $$R_{\text{extraction}}=\frac{N_{\text{detection}}}{N_{\text{reference}}}=\frac{\mathrm{TP}+\mathrm{FP}}{\mathrm{TP}+\mathrm{FN}}$$

(14) $$R_{\text{match}}=\frac{N_{\text{match}}}{N_{\text{reference}}}=\frac{\mathrm{TP}}{\mathrm{TP}+\mathrm{FN}}$$

(15) $$R_{\text{commission}}=\frac{N_{\text{commission}}}{N_{\text{detection}}}=\frac{\mathrm{FP}}{\mathrm{TP}+\mathrm{FP}}=1-\frac{R_{\text{match}}}{R_{\text{extraction}}}$$

(16) $$R_{\text{ommission}}=\frac{N_{\text{ommission}}}{N_{\text{reference}}}=\frac{\mathrm{FN}}{\mathrm{TP}+\mathrm{FN}}=1-R_{\text{match}}$$

(17) $$F=2\times \frac{R_{\text{match}}\times \frac{R_{\text{match}}}{R_{\text{extraction}}}}{R_{\text{match}}+\frac{R_{\text{match}}}{R_{\text{extraction}}}}$$

*R*_match_, *R*_extraction_, and *F* are the main assessment metrics, related to the overall accuracy, and the closer they are to 1, the higher the accuracy of the ITS algorithm. *R*_omission_ and *R*_commission_ are secondary assessment metrics to measure the degree of under‐ and over‐segmentation, and the closer they are to 0, the less under‐ or over‐segmentation. The above metrics are used for tree top detection, and tree height estimation is evaluated by coefficient of determination (*R* ^2^) and root mean square error (RMSE).

## 3 RESULTS

### 3.1 Treetop detection results

**FIGURE 6:**
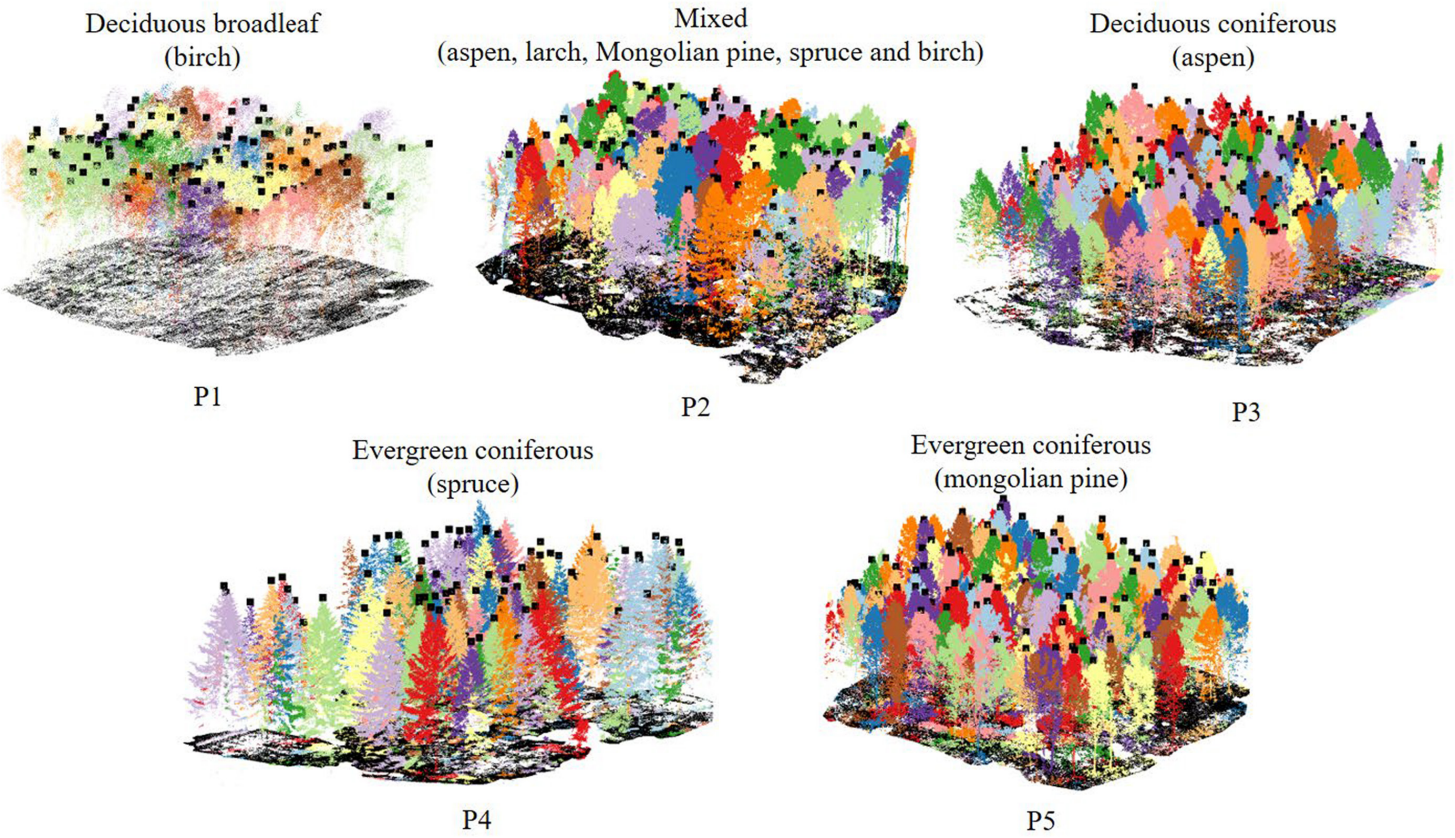
Diagram of the tree top detection of five plots in Saihanba. The black discrete points are the reference tree top coordinates, and the normalized point clouds with different colors are the ITS results.

Figure 6 shows our ITS results with corresponding reference tree top locations of the five plots located in Saihanba. The results of P2–P5 are visually pleasing and match well with the reference positions. However, for P1, it is difficult to evaluate the segmentation results because the tree tops of the broadleaf cannot be observed clearly on one hand, on the other hand, the point cloud density in this plot is relatively low.

**FIGURE 7:**
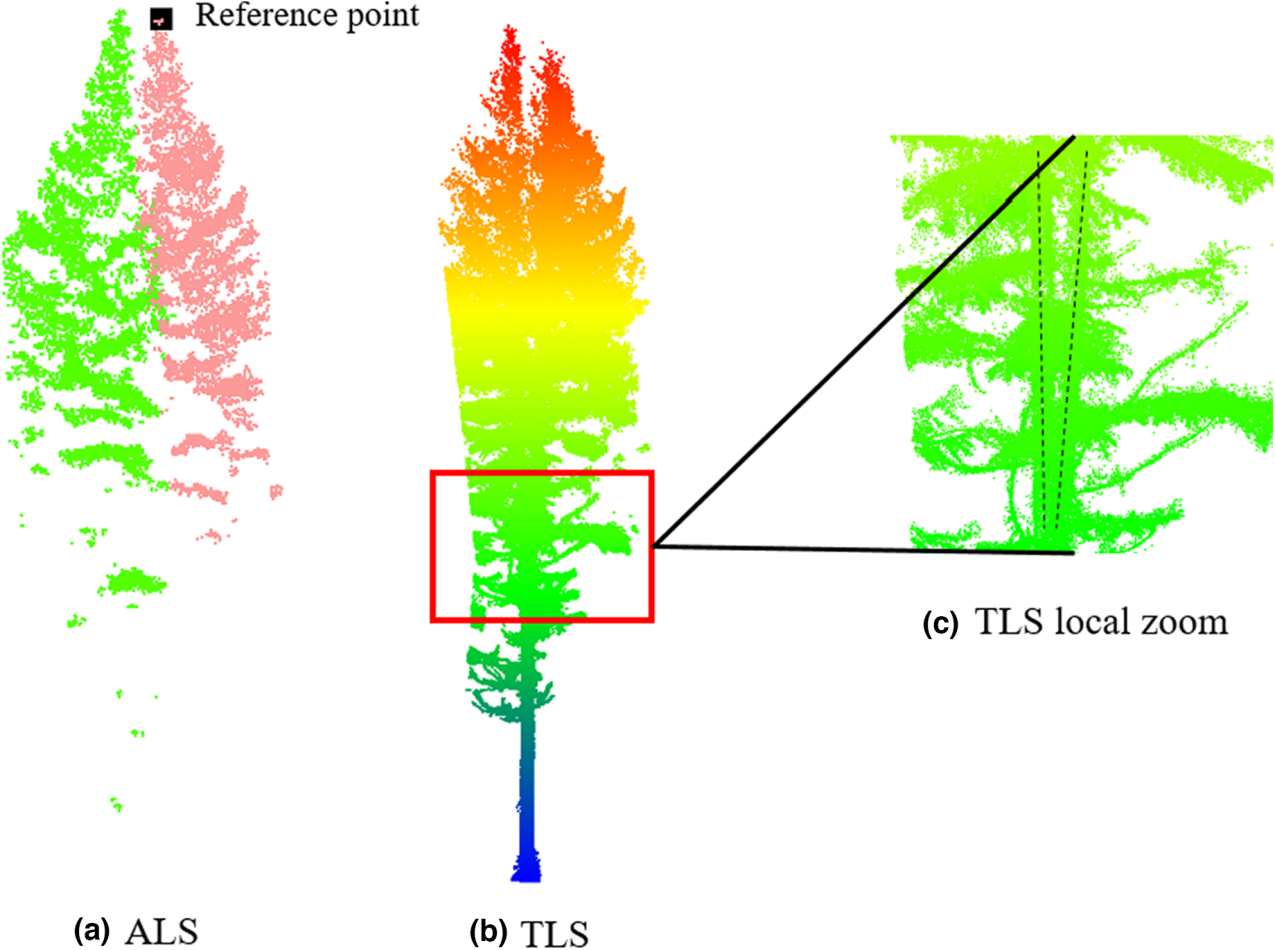
A case of trunk bifurcation of the larch tree in P3 which looks like two trees over the top of the tree. (a) is the ULS point clouds and (b) is the corresponding TLS LiDAR point clouds. (c) shows the local zoom of the TLS, where two similar bifurcations are depicted by dashed lines.

The quantitative assessment results are presented in Table 5. Overall, the segmentation accuracy is fine with an average match rate and *F*‐score greater than 0.7. However, there is some over‐segmentation, especially in P3 and P5 with a relatively lower *R*_omission_. We checked ULS and TLS data carefully and found that the conifers in Sahanba, especially the larch, are prone to trunk bifurcation. A case is shown in Figure 7 to illustrate the phenomenon of trunk bifurcation. The phenomenon can be clearly seen in the TLS point clouds (Figure 7b,c). However, the tree trunk is not clearly visible through the ULS point clouds due to the occlusion issue, which causes it to look similar to two trees (Figure 7a).

**TABLE 5:** Results of treetop detection of P1–P5 using our ITS algorithm.

Plot	*R*_match_	*R*_extraction_	*F*	*R*_omission_	*R*_commission_
P1	0.61	0.80	0.68	0.39	0.23
P2	0.75	1.24	0.67	0.25	0.39
P3	0.92	1.76	0.66	0.08	0.48
P4	0.71	0.92	0.74	0.29	0.23
P5	0.89	1.34	0.75	0.11	0.34
Average	0.78	1.21	0.70	0.22	0.33

### 3.2 Tree height accuracy evaluation

**FIGURE 8:**
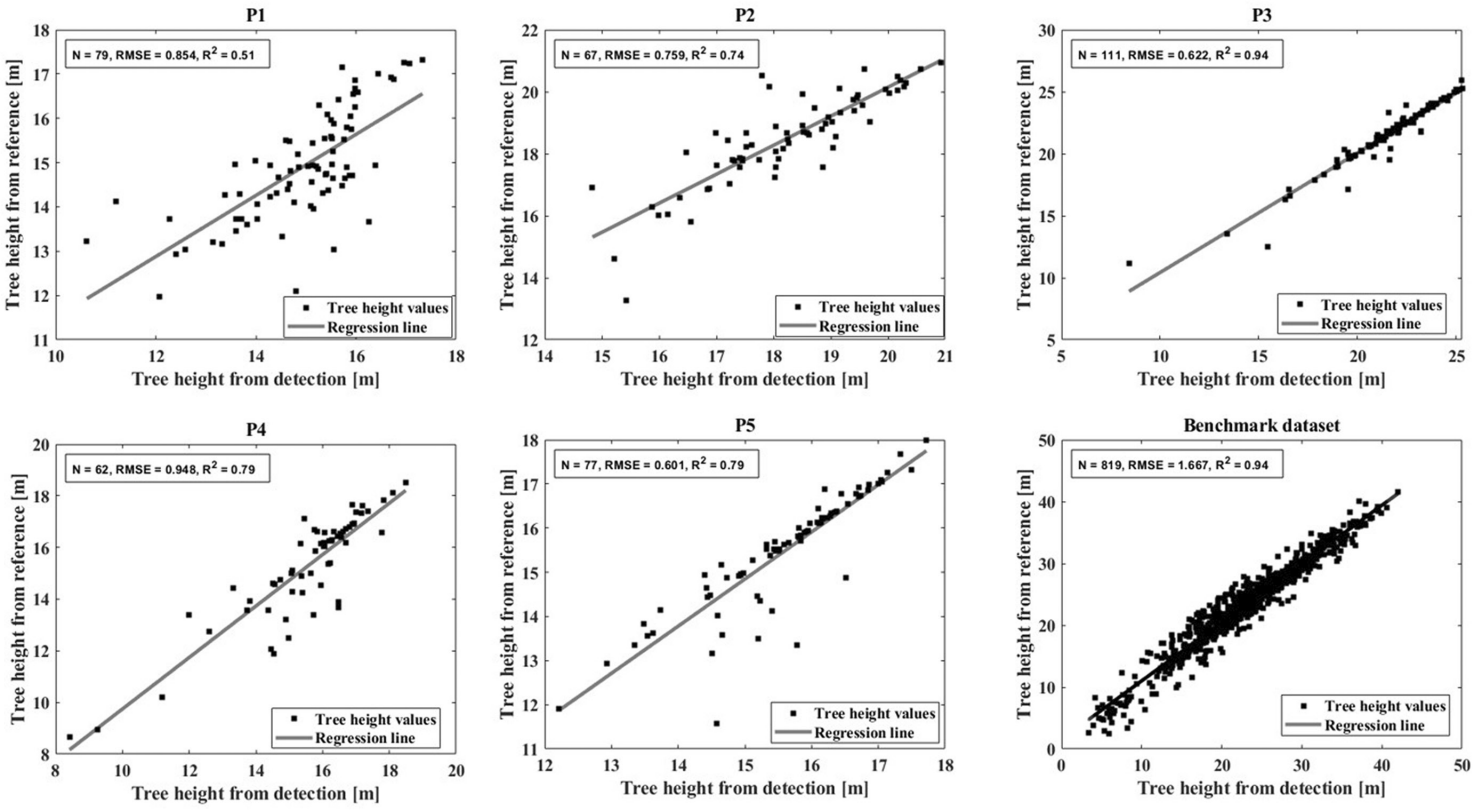
Tree height estimation results of P1–P5 and Benchmark dataset.

The accuracy of tree height extraction is evaluated by comparing the reference with the matched tree heights. As seen in Figure 8, all the results are well except for P1. P3 and the 14 plots of benchmark dataset are the best with *R* ^2^ = .94, although the RMSE of the benchmark dataset is 1.667 m. The results for P2, P4, and P5 are relatively well, with *R* ^2^ = .79 (.74 for P2) and RMSE < 1 m. In general, our method can accurately extract the tree height of coniferous and mixed forests. For broadleaf forests, especially on slopes, the precise extraction of tree height requires more effort.

### 3.3 Comparison with existing methods

To evaluate our approach more comprehensively, we choose three classical ITS methods for comparison, including the WS (Wang et al., 2004), mark‐controlled watershed (denoted as MCWS) (Chen et al., 2006), and point cloud region growing segmentation (denoted as PCS) (Li et al., 2012). The WS and PCS are implemented through the lidR tool (Roussel et al., 2020), and the MCWS implemented through Digital‐Forestry‐Toolbox (https://mparkan.github.io/Digital‐Forestry‐Toolbox/). Due to the high densities of the ULS point clouds in P2–P5, the PCS cannot be executed effectively. Therefore, only the results of P1 are available. For sample P2–5, we use CloudCompare software to subsample the point clouds for the PCS method. Table 6 shows the average ITS results of P1–P5. The matching rate, F‐score, and ommission rate of our algorithm are most well compared to the WS, MCWS and PCS. The results of the MCWS are extremely poor, which may be due to the parameter settings, and the reasons are analyzed in the Section 4. The results of tree top detection using the four different methods in P1 are shown in Table 7. Compared with the other three methods, our method gives the best results.

**TABLE 6:** Tree top detection results of P1–P5 using three different methods.

Method	*R*_match_	*R*_extraction_	*F*	*R*_omission_	*R*_commission_
WS	0.74	**1.11**	**0.70**	0.26	0.28
MCWS	0.34	0.48	0.46	0.66	0.16
PCS	0.57	0.58	**0.71**	0.43	**0.02**
Ours	**0.78**	1.21	0.70	**0.22**	0.33

Bolded values show optimal results.

**TABLE 7:** Tree top detection results of P1 using four different methods.

Method	*R*_match_	*R*_extraction_	*F*	*R*_omission_	*R*_commission_
WS	0.43	0.49	0.57	0.57	0.13
MCWS	0.20	0.20	0.33	0.80	**0.00**
PCS	0.38	0.38	0.55	0.62	**0.00**
Ours	**0.61**	**0.80**	**0.68**	**0.39**	0.23

Bolded values show optimal results.

**FIGURE 9:**
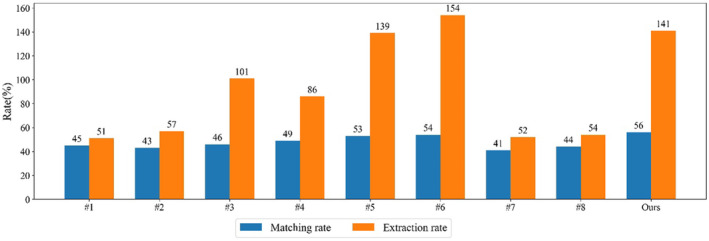
Tree top detection results of 14 public plots in Europe using our and other eight methods. #1–#8 correspond to the methods described by Eysn et al. (2015).

Table 8 and Figure 9 show the ITS results of 14 public plots in the benchmark dataset. Compared with the WS, MCWS, and PCS, our method gives the best matching rate. Although the *F*‐score by our algorithm is 0.02 lower than that by MCWS, our matching rate is 0.16 higher. Our method also gives the best matching rate compared to methods #1–#8 described by Eysn et al. (2015). Of these methods, WS, #5, #6, and ours matched more than 50%. All the four methods give over‐segmentation result, while ours is at the medium level.

**TABLE 8:** Tree top detection results of 14 public plots in Europe using four different methods.

Method	*R*_match_	*R*_extraction_	*F*	*R*_omission_	*R*_commission_
WS	0.52	**1.28**	0.47	0.48	0.47
MCWS	0.21	0.35	0.28	0.79	0.23
PCS	0.38	0.50	**0.50**	0.62	**0.19**
Ours	**0.56**	1.41	0.48	**0.44**	0.50

Bolded values show optimal results.

## 4 DISCUSSION

### 4.1 Sensitivity analysis and parameter settings

**FIGURE 10:**
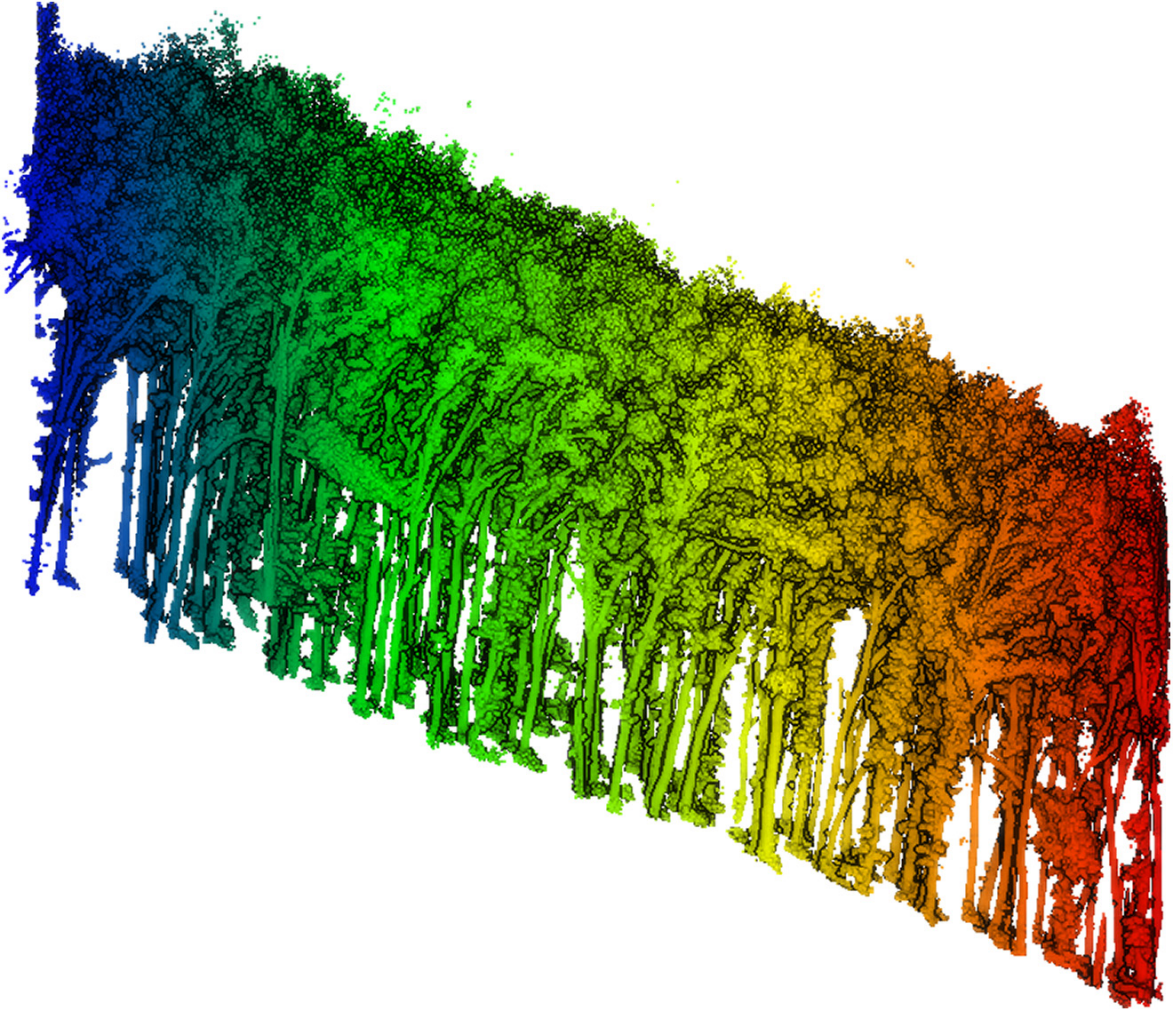
Side view of the TLS point cloud data of P1.

For P1, the result is relatively poor with the *R* ^2^ = .5. There are three reasons for this: (1) the average slope of this plot is 30°, so the point cloud normalization will cause distortion of the trees (Khosravipour et al., 2015). (2) there is distortion of the trunk of birch due to the natural environment; (3) there is no obvious top of broadleaf trees, which is different from coniferous trees. So it is difficult to accurately detect tree tops even visually. The above factors cause errors in both field measurements and algorithm estimation. Figure 10 shows the TLS point cloud data of P1 and clearly confirms the three analyses above.

P1 was the most complex plot in this study, with complex topographic conditions, the highest tree stem density, irregular canopy shape, and relatively low point cloud density. Therefore, it was used for the sensitivity and parameter settings analysis. For the optimized CCE, the optimal clustering scale is determined by Equations ((10), (11), (12)). With Equation (10), it is ensured that the shape of the canopy is reasonable and unreasonably flattened canopy is removed. With Equations (11) and (12), the distance between the top and edge is determined by projection in two directions, and then, the minimum distance threshold is set to ensure that the top is located near the center of the canopy. Table 9 demonstrates the effect of the minimum distance threshold setting on the results in P1. If no minimum distance is set (or a small value, e.g., 1/16 crown diameter), over‐segmentation will be very serious. However, if this threshold is set too large (e.g., 1/4 crown diameter), many trees will not be segregated, especially for broadleaf forests with inconspicuous tree tops. Therefore, this threshold was set to 1/8 crown diameter to ensure its applicability in both coniferous and broadleaf forests.

**TABLE 9:** Setting of the minimum distance threshold from the top to the edge for the trees in P1.

Top‐edge min. distance	*R*_match_	*R*_extraction_	*F*	*R*_omission_	*R*_commission_
1/4 crown diameter	0.44	0.51	0.59	0.56	**0.13**
1/8 crown diameter	0.61	**0.80**	**0.68**	0.39	0.23
1/16 crown diameter	0.79	1.39	0.66	0.21	0.43
No limitation	**0.95**	1.99	0.64	**0.05**	0.52

Bolded values show optimal results.

**FIGURE 11:**
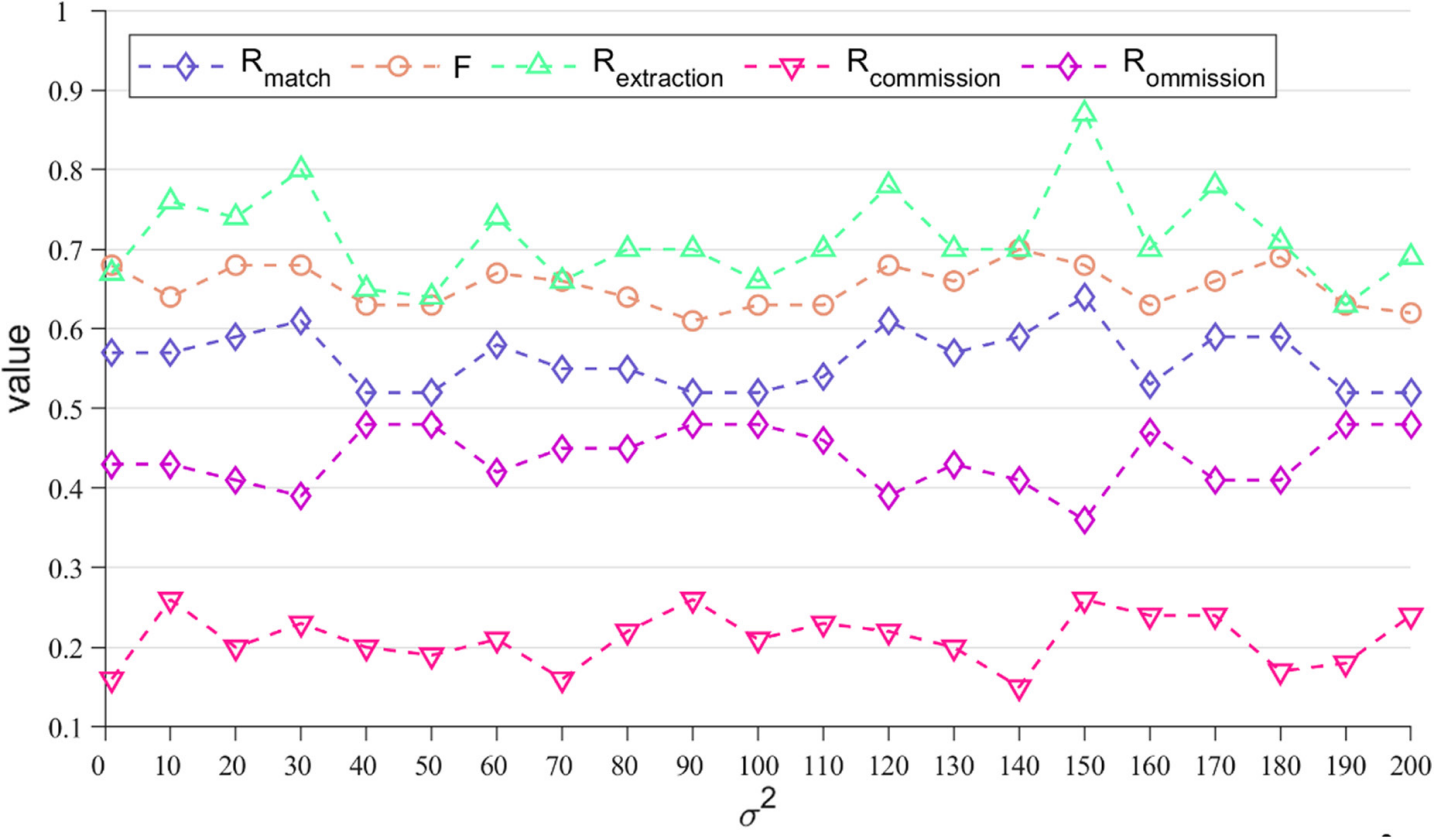
Variations of the tree top detection assessment metrics with *σ* ^2^.

There are two input parameters in our algorithm. The vertical distance correction factor, *Vr*, is to be considered for ULS/ALS point cloud clustering. In our study, *Vr* is set to 1/6 according to the best results given by Pang et al. (2021). The empirical coefficient related to the Gaussian kernel, *σ*, is was analyzed in our study. The variation of extraction rate, matching rate, commission rate, omission rate, and *F* score with *σ* ^2^ is shown in Figure 11. It can be seen that these five evaluation metrics are very stable, indicating that our algorithm is robust.

To fairly compare various ITS methods, the same canopy structure related parameters were set in all test plots (Table 10). These parameters are either program default parameters or determined by reference to previous studies. For the parameter related to the point cloud density, that is, the resolution of the CHM, we set this parameter to 0.2 m for ALS generation and 0.1 m for ULS generation. For the MCWS, the relationship between tree height and canopy radius is required. However, field measurements are difficult to obtain sufficient accuracy and enough data, so we refer to the formulas by Popescu and Wynne (2004) (See Table 10). In the previous section, the MCWS gave poor results in many plots. This is due to the inappropriate relationship between the tree height and crown radius within the plots, and not the algorithm itself. The properties of different types of trees should be complex, but due to field measurements constraints, only three fixed formulas are given for broadleaf, coniferous, and mixed forests (Popescu & Wynne, 2004).

**TABLE 10:** Parameter setting in different methods used for comparison. *h* stands for tree height.

Algorithms	Parameters	Values	Explanation
PCS	Zu	15 [m]	Height threshold
	dt1	1.5 [m]	Spacing threshold when tree height > Zu
	dt2	2 [m]	Spacing threshold when tree height < Zu
	*R*	2 [m]	Search radius
WS	Tolerance	1 [m]	The min height of a tree between its top and another tree
	Ext	2 [pixel]	The radius of the neighborhood in pixels for detection of neighboring
MCWS	Search radius	(3.09632 + 0.00895 * *h* ^2^)/2	Deciduous forest
		(3.75105–0.17919 * *h* + 0.01241 * *h* ^2^)/2	Coniferous forests
		(2.51503 + 0.00901 * *h* ^2^)/2	Mixed forests
Ours	*Vr*	1/6	Vertical distance correction factor
	*σ*	$\sqrt{30}$	Gaussian kernel

### 4.2 Efficient implementation

With the development of LiDAR hardware technology, high quality and density ULS/ALS LiDAR point clouds are emerging. Therefore, ITS algorithms are also expected to be able to process data efficiently. Thanks to the initial segmentation using the watershed and the mean shift voxelization, the execution speed of the CCE algorithm has been greatly improved. The processing speed of the improved CCE algorithm was tested with the configuration of a Core Intel(R) Core(TM) i7‐8700 CPU@3.20GHz Processor, 40 GB RAM, an NVIDIA GeForce GTX 1660 graphics card and the Microsoft Windows 10 operating system. The results are shown in Table 11. We did not compare the original CCE algorithm because there was not enough memory in the device we used for the method to run successfully. For the P3 with 50 × 50 m with a density of over 1500 pts/m^2^, the time to run the algorithm is within 6 min. Our ITS method has the potential to meet the upcoming era of massive point clouds.

**TABLE 11:** Program runtime in different plots.

Plot	P1	P2	P3	P4	P5
Point density(pts/m^2^)	298	3295	1636	1473	3976
Size (m^2^)	900	900	2500	900	900
Cost time (s)	**8.439**	**192.340**	**357.954**	**87.199**	**231.257**

Bolded values show optimal results.

### 4.3 Future prospects

By segmenting the ULS/ALS point clouds, each tree coordinates, height, and crown width can be further extracted. The open source code we provide already enables this function. The accuracy of tree top detection and tree height extraction has been demonstrated and discussed in this study. However, the accuracy of crown width estimation is lacking. This is due to the difficulty in finding a valid and accurate method for crown width measurement. We have tried to manually extract the crown width of each tree from the TLS LiDAR point clouds. However, this attempt failed because it was so time‐consuming and labor‐intensive, and in many cases, it was impossible to distinguish each tree manually. Perhaps in the future, the enhancement of TLS ITS algorithms and open source of the code will facilitate the research of crown width estimation. In addition, the method process can be further optimized in the future, for example, tree‐top detection is added to the CCE method. Currently, we have not found a method that can handle complex‐shaped canopies on a large scale. Therefore, we use the simple qualifications of Equations ((10), (11), (12)). In the future, it is necessary to improve the accuracy in complex forest and terrain conditions.

## 5 CONCLUSION

Individual tree segmentation using ALS or ULS data is still a challenge due to the complexity of forest structure. In this paper, we proposed a new individual tree segmentation method, which consists of the WS algorithm, and the optimized CCE algorithm. We optimized the CCE algorithm to make it more efficient, and the optimal segmentation scale can be determined automatically by taking into account the structural characteristics of the canopy. The new ITS method can take full advantages of the efficient of the WS and the accuracy of CCE algorithm. Additionally, the new method is robust for the complex plots and insensitive for the parameters. Tree coordinates and heights are extracted and output directly automatically.

Validation at five different forest types of plots in China and 14 public plots in Europe showed the accuracy of both treetop detection and tree height estimation. Compared with the other 11 individual tree segmentation methods, our method gives better results. Through sensitivity analysis for input parameters, we find that the algorithm is robust. Efficient processing speed enables it to meet the high‐density point clouds of 4000 pts/m^2^. Our method is both practical and applicable and can be used to extract the structural parameters of individual trees over large areas for forest management, carbon stock estimation, and habitat mapping.

## AUTHOR CONTRIBUTIONS

**Yi Li:** Conceptualization (lead); data curation (lead); formal analysis (lead); methodology (lead); validation (lead); writing – original draft (lead). **Donghui Xie:** Conceptualization (equal); funding acquisition (equal); methodology (equal); project administration (lead); supervision (lead); writing – review and editing (lead). **Yingjie Wang:** Supervision (equal); writing – review and editing (equal). **Shuangna Jin:** Investigation (equal); writing – review and editing (equal). **Kun Zhou:** Validation (equal); writing – review and editing (equal). **Zhixiang Zhang:** Validation (equal); writing – review and editing (equal). **Weihua Li:** Validation (equal); writing – review and editing (equal). **Wuming Zhang:** Validation (equal); writing – review and editing (equal). **Xihan Mu:** Supervision (equal); writing – review and editing (equal). **Guangjian Yan:** Funding acquisition (lead); supervision (equal); writing – review and editing (lead).

## CONFLICT OF INTEREST STATEMENT

We declare no conflicts of interest with this research.

## Data Availability

The source code can be downloaded freely from https://github.com/liyi‐rs/ITS_WS_ICCE. Benchmark dataset can be available from the NEWFOR website (https://www.newfor.net/).

## References

[ece310297-bib-0001] Axelsson, P. (2000). DEM generation from laser scanner data using adaptive TIN models. International Archives of Photogrammetry and Remote Sensing, 33, 110–117. 10.1016/j.isprsjprs.2005.10.005

[ece310297-bib-0002] Ayrey, E. , Fraver, S. , Kershaw, J. A., Jr. , Kenefic, L. S. , Hayes, D. , Weiskittel, A. R. , & Roth, B. E. (2017). Layer stacking: A novel algorithm for individual forest tree segmentation from LiDAR point clouds. Canadian Journal of Remote Sensing, 43, 16–27. 10.1080/07038992.2017.1252907

[ece310297-bib-0003] Balsi, M. , Esposito, S. , Fallavollita, P. , & Nardinocchi, C. (2018). Single‐tree detection in high‐density LiDAR data from UAV‐based survey. European Journal of Remote Sensing, 51, 679–692. 10.1080/22797254.2018.1474722

[ece310297-bib-0004] Burt, A. , Disney, M. , & Calders, K. (2019). Extracting individual trees from lidar point clouds using treeseg. Methods in Ecology and Evolution, 10, 438–445. 10.1111/2041-210X.13121

[ece310297-bib-0005] Chen, Q. , Baldocchi, D. , Gong, P. , & Kelly, M. (2006). Isolating individual trees in a savanna woodland using small footprint lidar data. Photogrammetric Engineering & Remote Sensing, 72, 923–932. 10.14358/Pers.72.8.923

[ece310297-bib-0006] Comaniciu, D. , & Meer, P. (2002). Mean shift: A robust approach toward feature space analysis. IEEE Transactions on Pattern Analysis and Machine Intelligence, 24, 603–619. 10.1109/34.1000236

[ece310297-bib-0007] Dai, W. , Yang, B. , Dong, Z. , & Shaker, A. (2018). A new method for 3D individual tree extraction using multispectral airborne LiDAR point clouds. ISPRS Journal of Photogrammetry and Remote Sensing, 144, 400–411. 10.1016/j.isprsjprs.2018.08.010

[ece310297-bib-0008] Dalponte, M. , & Coomes, D. A. (2016). Tree‐centric mapping of forest carbon density from airborne laser scanning and hyperspectral data. Methods in Ecology and Evolution, 7, 1236–1245. 10.1111/2041-210X.12575 28008347 PMC5137341

[ece310297-bib-0009] Eysn, L. , Hollaus, M. , Lindberg, E. , Berger, F. , Monnet, J.‐M. , Dalponte, M. , Kobal, M. , Pellegrini, M. , Lingua, E. , & Mongus, D. (2015). A benchmark of lidar‐based single tree detection methods using heterogeneous forest data from the alpine space. Forests, 6, 1721–1747. 10.3390/f6051721

[ece310297-bib-0010] Geng, X. , & Tang, H. (2020). Clustering by connection center evolution. Pattern Recognition, 98, 107063. 10.1016/j.patcog.2019.107063

[ece310297-bib-0011] Guo, Q. , Su, Y. , Hu, T. , Guan, H. , Jin, S. , Zhang, J. , Zhao, X. , Xu, K. , Wei, D. , & Kelly, M. (2020). Lidar boosts 3D ecological observations and modelings: A review and perspective. IEEE Geoscience and Remote Sensing Magazine, 9, 232–257. 10.1109/MGRS.2020.3032713

[ece310297-bib-0012] Hu, B. , Li, J. , Jing, L. , & Judah, A. (2014). Improving the efficiency and accuracy of individual tree crown delineation from high‐density LiDAR data. International Journal of Applied Earth Observation and Geoinformation, 26, 145–155. 10.1016/j.jag.2013.06.003

[ece310297-bib-0013] Hyyppa, J. , Kelle, O. , Lehikoinen, M. , & Inkinen, M. (2001). A segmentation‐based method to retrieve stem volume estimates from 3‐D tree height models produced by laser scanners. IEEE Transactions on Geoscience and Remote Sensing, 39, 969–975. 10.1109/36.921414

[ece310297-bib-0014] Jaskierniak, D. , Lucieer, A. , Kuczera, G. , Turner, D. , Lane, P. , Benyon, R. , & Haydon, S. (2021). Individual tree detection and crown delineation from Unmanned Aircraft System (UAS) LiDAR in structurally complex mixed species eucalypt forests. ISPRS Journal of Photogrammetry and Remote Sensing, 171, 171–187. 10.1016/j.isprsjprs.2020.10.016

[ece310297-bib-0015] Jing, L. , Hu, B. , Li, J. , & Noland, T. (2012). Automated delineation of individual tree crowns from LiDAR data by multi‐scale analysis and segmentation. Photogrammetric Engineering & Remote Sensing, 78, 1275–1284. 10.14358/PERS.78.11.1275

[ece310297-bib-0016] Katoh, M. , & Gougeon, F. A. (2012). Improving the precision of tree counting by combining tree detection with crown delineation and classification on homogeneity guided smoothed high resolution (50 cm) multispectral airborne digital data. Remote Sensing, 4, 1411–1424. 10.3390/rs4051411

[ece310297-bib-0017] Kellner, J. R. , Armston, J. , Birrer, M. , Cushman, K. , Duncanson, L. , Eck, C. , Falleger, C. , Imbach, B. , Král, K. , & Krůček, M. (2019). New opportunities for forest remote sensing through ultra‐high‐density drone lidar. Surveys in Geophysics, 40, 959–977. 10.1007/s10712-019-09529-9 31395993 PMC6647463

[ece310297-bib-0018] Khosravipour, A. , Skidmore, A. K. , Isenburg, M. , Wang, T. , & Hussin, Y. A. (2014). Generating pit‐free canopy height models from airborne lidar. Photogrammetric Engineering & Remote Sensing, 80, 863–872. 10.14358/Pers.80.9.863

[ece310297-bib-0019] Khosravipour, A. , Skidmore, A. K. , Wang, T. , Isenburg, M. , & Khoshelham, K. (2015). Effect of slope on treetop detection using a LiDAR canopy height model. ISPRS Journal of Photogrammetry and Remote Sensing, 104, 44–52. 10.1016/j.isprsjprs.2015.02.013

[ece310297-bib-0020] Korhonen, L. , Packalen, P. , & Rautiainen, M. (2017). Comparison of Sentinel‐2 and Landsat 8 in the estimation of boreal forest canopy cover and leaf area index. Remote Sensing of Environment, 195, 259–274. 10.1016/j.rse.2017.03.021

[ece310297-bib-0021] Leckie, D. G. , Gougeon, F. A. , Tinis, S. , Nelson, T. , Burnett, C. N. , & Paradine, D. (2005). Automated tree recognition in old growth conifer stands with high resolution digital imagery. Remote Sensing of Environment, 94, 311–326. 10.1016/j.rse.2004.10.011

[ece310297-bib-0022] Lefsky, M. A. , Cohen, W. B. , Parker, G. G. , & Harding, D. J. (2002). Lidar remote sensing for ecosystem studies: Lidar, an emerging remote sensing technology that directly measures the three‐dimensional distribution of plant canopies, can accurately estimate vegetation structural attributes and should be of particular interest to forest, landscape, and global ecologists. Bioscience, 52, 19–30. 10.1641/0006-3568(2002)052[0019:LRSFES]2.0.CO;2

[ece310297-bib-0023] Li, W. , Guo, Q. , Jakubowski, M. K. , & Kelly, M. (2012). A new method for segmenting individual trees from the lidar point cloud. Photogrammetric Engineering & Remote Sensing, 78, 75–84. 10.14358/Pers.78.1.75

[ece310297-bib-0024] Liang, J. , Crowther, T. W. , Picard, N. , Wiser, S. , Zhou, M. , Alberti, G. , Schulze, E.‐D. , McGuire, A. D. , Bozzato, F. , & Pretzsch, H. (2016). Positive biodiversity‐productivity relationship predominant in global forests. Science, 354, aaf8957. 10.1126/science.aaf8957 27738143

[ece310297-bib-0025] Lindberg, E. , Eysn, L. , Hollaus, M. , Holmgren, J. , & Pfeifer, N. (2014). Delineation of tree crowns and tree species classification from full‐waveform airborne laser scanning data using 3‐D ellipsoidal clustering. IEEE Journal of Selected Topics in Applied Earth Observations and Remote Sensing, 7, 3174–3181. 10.1109/Jstars.2014.2331276

[ece310297-bib-0026] Lindberg, E. , & Holmgren, J. (2017). Individual tree crown methods for 3D data from remote sensing. Current Forestry Reports, 3, 19–31. 10.1007/s40725-017-0051-6

[ece310297-bib-0027] Lu, X. , Guo, Q. , Li, W. , & Flanagan, J. (2014). A bottom‐up approach to segment individual deciduous trees using leaf‐off lidar point cloud data. ISPRS Journal of Photogrammetry and Remote Sensing, 94, 1–12. 10.1016/j.isprsjprs.2014.03.014

[ece310297-bib-0028] Pang, Y. , Li, Z. , Ju, H. , Lu, H. , Jia, W. , Si, L. , Guo, Y. , Liu, Q. , Li, S. , & Liu, L. (2016). LiCHy: The CAF's LiDAR, CCD and hyperspectral integrated airborne observation system. Remote Sensing, 8, 398. 10.3390/rs8050398

[ece310297-bib-0029] Pang, Y. , Wang, W. , Du, L. , Zhang, Z. , Liang, X. , Li, Y. , & Wang, Z. (2021). Nyström‐based spectral clustering using airborne LiDAR point cloud data for individual tree segmentation. International Journal of Digital Earth, 14, 1452–1476. 10.1080/17538947.2021.1943018

[ece310297-bib-0030] Popescu, S. C. , & Wynne, R. H. (2004). Seeing the trees in the forest. Photogrammetric Engineering & Remote Sensing, 70, 589–604. 10.14358/PERS.70.5.589

[ece310297-bib-0031] Reitberger, J. , Schnörr, C. , Krzystek, P. , & Stilla, U. (2009). 3D segmentation of single trees exploiting full waveform LIDAR data. ISPRS Journal of Photogrammetry and Remote Sensing, 64, 561–574. 10.1016/j.isprsjprs.2009.04.002

[ece310297-bib-0032] Roussel, J.‐R. , Auty, D. , Coops, N. C. , Tompalski, P. , Goodbody, T. R. , Meador, A. S. , Bourdon, J.‐F. , de Boissieu, F. , & Achim, A. (2020). lidR: An R package for analysis of Airborne Laser Scanning (ALS) data. Remote Sensing of Environment, 251, 112061. 10.1016/j.rse.2020.112061

[ece310297-bib-0033] Seidl, R. , Thom, D. , Kautz, M. , Martin‐Benito, D. , Peltoniemi, M. , Vacchiano, G. , Wild, J. , Ascoli, D. , Petr, M. , & Honkaniemi, J. (2017). Forest disturbances under climate change. Nature Climate Change, 7, 395–402. 10.1038/Nclimate3303 PMC5572641 28861124

[ece310297-bib-0034] Solberg, S. , Naesset, E. , & Bollandsas, O. M. (2006). Single tree segmentation using airborne laser scanner data in a structurally heterogeneous spruce forest. Photogrammetric Engineering & Remote Sensing, 72, 1369–1378. 10.14358/Pers.72.12.1369

[ece310297-bib-0035] Strîmbu, V. F. , & Strîmbu, B. M. (2015). A graph‐based segmentation algorithm for tree crown extraction using airborne LiDAR data. ISPRS Journal of Photogrammetry and Remote Sensing, 104, 30–43. 10.1016/j.isprsjprs.2015.01.018

[ece310297-bib-0036] Tao, S. , Wu, F. , Guo, Q. , Wang, Y. , Li, W. , Xue, B. , Hu, X. , Li, P. , Tian, D. , & Li, C. (2015). Segmenting tree crowns from terrestrial and mobile LiDAR data by exploring ecological theories. ISPRS Journal of Photogrammetry and Remote Sensing, 110, 66–76. 10.1016/j.isprsjprs.2015.10.007

[ece310297-bib-0037] Tochon, G. , Feret, J.‐B. , Valero, S. , Martin, R. E. , Knapp, D. E. , Salembier, P. , Chanussot, J. , & Asner, G. P. (2015). On the use of binary partition trees for the tree crown segmentation of tropical rainforest hyperspectral images. Remote Sensing of Environment, 159, 318–331. 10.1016/j.rse.2014.12.020

[ece310297-bib-0038] Vauhkonen, J. , Ene, L. , Gupta, S. , Heinzel, J. , Holmgren, J. , Pitkänen, J. , Solberg, S. , Wang, Y. , Weinacker, H. , & Hauglin, K. M. (2012). Comparative testing of single‐tree detection algorithms under different types of forest. Forestry, 85, 27–40. 10.1093/forestry/cpr051

[ece310297-bib-0039] Véga, C. , Hamrouni, A. , El Mokhtari, S. , Morel, J. , Bock, J. , Renaud, J.‐P. , Bouvier, M. , & Durrieu, S. (2014). PTrees: A point‐based approach to forest tree extraction from lidar data. International Journal of Applied Earth Observation and Geoinformation, 33, 98–108. 10.1016/j.jag.2014.05.001

[ece310297-bib-0040] Wallace, L. , Lucieer, A. , & Watson, C. S. (2014). Evaluating tree detection and segmentation routines on very high resolution UAV LiDAR data. IEEE Transactions on Geoscience and Remote Sensing, 52, 7619–7628. 10.1109/TGRS.2014.2315649

[ece310297-bib-0041] Wang, L. , Gong, P. , & Biging, G. S. (2004). Individual tree‐crown delineation and treetop detection in high‐spatial‐resolution aerial imagery. Photogrammetric Engineering & Remote Sensing, 70, 351–357.

[ece310297-bib-0042] Wang, Y. , Hyyppä, J. , Liang, X. , Kaartinen, H. , Yu, X. , Lindberg, E. , Holmgren, J. , Qin, Y. , Mallet, C. , & Ferraz, A. (2016). International benchmarking of the individual tree detection methods for modeling 3‐D canopy structure for silviculture and forest ecology using airborne laser scanning. IEEE Transactions on Geoscience and Remote Sensing, 54, 5011–5027. 10.1109/TGRS.2016.2543225

[ece310297-bib-0043] Williams, J. , Schönlieb, C.‐B. , Swinfield, T. , Lee, J. , Cai, X. , Qie, L. , & Coomes, D. A. (2019). 3D segmentation of trees through a flexible multiclass graph cut algorithm. IEEE Transactions on Geoscience and Remote Sensing, 58, 754–776. 10.1109/TGRS.2019.2940146

[ece310297-bib-0044] Yang, Q. , Su, Y. , Jin, S. , Kelly, M. , Hu, T. , Ma, Q. , Li, Y. , Song, S. , Zhang, J. , & Xu, G. (2019). The influence of vegetation characteristics on individual tree segmentation methods with airborne LiDAR data. Remote Sensing, 11, 2880. 10.3390/rs11232880

[ece310297-bib-0045] Yin, D. , & Wang, L. (2019). Individual mangrove tree measurement using UAV‐based LiDAR data: Possibilities and challenges. Remote Sensing of Environment, 223, 34–49. 10.1016/j.rse.2018.12.034

[ece310297-bib-0046] Yun, T. , Jiang, K. , Li, G. , Eichhorn, M. P. , Fan, J. , Liu, F. , Chen, B. , An, F. , & Cao, L. (2021). Individual tree crown segmentation from airborne LiDAR data using a novel Gaussian filter and energy function minimization‐based approach. Remote Sensing of Environment, 256, 112307. 10.1016/j.rse.2021.112307

[ece310297-bib-0047] Zhang, W. , Cai, S. , Liang, X. , Shao, J. , Hu, R. , Yu, S. , & Yan, G. (2020). Cloth simulation‐based construction of pit‐free canopy height models from airborne LiDAR data. Forest Ecosystems, 7, 1–13. 10.1186/s40663-019-0212-0

[ece310297-bib-0048] Zhang, W. , Qi, J. , Wan, P. , Wang, H. , Xie, D. , Wang, X. , & Yan, G. (2016). An easy‐to‐use airborne LiDAR data filtering method based on cloth simulation. Remote Sensing, 8, 501. 10.3390/rs8060501

[ece310297-bib-0049] Zhen, Z. , Quackenbush, L. J. , & Zhang, L. (2016). Trends in automatic individual tree crown detection and delineation—Evolution of LiDAR data. Remote Sensing, 8, 333. 10.3390/rs8040333

[ece310297-bib-0050] Zheng, Z. , Zeng, Y. , Schneider, F. D. , Zhao, Y. , Zhao, D. , Schmid, B. , Schaepman, M. E. , & Morsdorf, F. (2021). Mapping functional diversity using individual tree‐based morphological and physiological traits in a subtropical forest. Remote Sensing of Environment, 252, 112170. 10.1016/j.rse.2020.112170

